# Improving mental arithmetic ability of primary school students with schema teaching method: An experimental study

**DOI:** 10.1371/journal.pone.0297013

**Published:** 2024-04-16

**Authors:** Dawei Liu, Xin Tan, Huifen Yan, Wei Li

**Affiliations:** 1 College of Teacher Education, Hubei University of Education, Wuhan, China; 2 College of Mathematics and Statistics, Hubei University of Education, Wuhan, China; 3 Brain Science and Learning Science Committee ofs Hubei Teachers Education Association, Wuhan, China; Julius-Maximilians-Universität Würzburg, GERMANY

## Abstract

Skillful utilization of mental arithmetic can significantly improve students’ mathematical computation ability. However, it was observed that primary school students often resort to reiterating the process of written arithmetic in their minds during mental arithmetic, which is not conducive to their numerical ability improvement. This paper devises a set of graphic teaching aids for primary school students’ mental arithmetic improvement based on mental arithmetic strategies, schema theory, and working memory. To validate the effectiveness of schema teaching in enhancing mental arithmetic ability among primary school students, a controlled experiment was conducted with two groups of third-grade students randomly selected from a primary school in Jingshan City. The results, obtained through descriptive statistical analysis and the multitrait—multimethod approach (MTMM), indicated that the experimental group (n = 52) demonstrated significant improvements in speed, accuracy, and stability in mental addition and subtraction after a 14-day instruction period in schema teaching. This study offers a potent mental arithmetic teaching strategy for elementary mathematics education, which can lead to a comprehensive enhancement of students’ mental calculation abilities. It also holds promise for inspiring innovative teaching methodologies in primary and secondary mathematics education in the future.

## 1. Introduction

Numerical ability is fundamental to math learning. However, primary school students often perform poorly in the calculation, which is very likely to cause a series of problems. For example, in classroom teaching, slow calculation speed or low correct rate of students may not only affect the quality of individual mathematics learning, but also leads to a slower teaching pace of the overall teaching, which in turn affects the learning quality of the entire class. Richard Cowan (2011) pointed out that there is a high correlation between the calculation skills of simple addition and subtraction and the performance of mathematics among primary school students. In addition, numerical ability differs from mathematical ability in that the former can extend the application of mathematics to everyday situations, such as the counting of items in production activities, the sale of goods, and purchasing. And good numerical skills are very helpful in accomplishing these tasks [1].

Although “numerical skills” herein are discussed in the context of primary-school mathematics teaching, the teaching of it is not just a separate part from the teaching of mathematics, but rather an integral reflection on the ways in which mathematics is learned and its application to students’ lives. However, math teachers in Chinese primary schools often resort to simply explaining arithmetic rules and over-intensifying students’ calculation practice in an attempt to make their students’ numerical skills stand out through excessive exercises, especially in rural and township schools. McIntosh (2002) points out that traditional methods for numerical teaching are not sufficient to develop the numerical skills of primary school students in lower grades, and some of them may even receive counterproductive results. In the mathematics teaching process of these schools, mathematics is perceived as an arithmetic course with no meaningful pedagogical method, a view that has existed for decades [2, 3].

LG Guthrie (2015) points out that when faced with arithmetic tasks, people often reorganize their physical displays by interacting with the environment, for example, perhaps moving coins around while counting money, taking notes with a pen, or gesturing, pointing or counting with their hands [4]. Obviously, all of these numerical behaviors affect calculation speed and correctness to varying degrees. But there is a more appropriate way, mental arithmetic. Mental arithmetic is a calculation method that relies on thinking and memory to arrive at an answer directly without the use of external tools, i.e., "oral arithmetic" commonly referred to. And it is an important thinking activity in daily activities [5], and closely related to working memory [6].

Existing research has found that multi-digit mental arithmetic plays an important role in teaching children how numbers work [7], how to decide on procedures, and how to create different strategies to solve math problems and promote a better understanding of number structure and its properties [8]. This body of research centers around the value of mental arithmetic skills to a child’s development, yet there is little current research on how to improve mental arithmetic skills. James R. Olsen (2015) also noted in his study that there are various reasons for this, for instance, mental arithmetic is often perceived to be based on an individual’s innate abilities, or there is no effective method to teach it but rigid extensive practice, with limited advancement as result [8, 9].

Based on the theory of mental arithmetic strategy and the schema theory, this paper proposes a schema teaching method, which utilizes its unique coding advantages and is supplemented by targeted explanations for mental arithmetic training. The schema teaching method is characterized by intuitive operation, which allows students to actively recognize, think and construct codes in the process of operation. In order to test its effectiveness, this paper will use the experimental method to compare the advantages of the schema teaching method and the traditional teaching method in improving the mental arithmetic ability of primary school students.

## 2. Schema teaching method

### 2.1. Mental arithmetic strategies

A strategy comprises procedures aimed at achieving higher-level goals or tasks [10]. Generally, primary school students can employ two math calculation strategies: written and mental calculations. The written calculation involves using a pen on paper to achieve an answer, while mental arithmetic is performing operations mentally without external tools [11]. Mental arithmetic strategies entail computational procedures without external tools, relying on thought and memory [12]. They are of great significance to proficiency and personal performance in mental calculation.

The mental arithmetic strategies used by primary school students can be broadly categorized into three types: N10, 1010, and pen-and-paper mental image. In the N10 strategy, the second number in the expression of an addition/subtraction problem is divided into units and tens, which are then added or subtracted; whereas the 1010 strategy divides the two numbers into units, adds/subtracts units with tens, then reassembles the final result [13]. Pen-and-paper mental image strategy reflects that written calculation mentally, which is considered to be inefficient [14].

Facing varied calculations, primary students progress from counting to written, then combined calculations. In China, addition, subtraction, multiplication, and division are performed in vertical form with all calculations coming down to mental arithmetic up to 10 finally. Thus the higher the grade, the more mental arithmetic is emphasized [15]. And more research focuses on mental arithmetic’s impact on students’ success.

According to Campbell (1987), the general framework for understanding multiplication fact retrieval in speeded arithmetic posits two parallel processes: a fact-retrieval process based on memory, and a calculation-based process that employs algorithmic procedures. Both processes operate in parallel and are in competition with each other. In the context of multiplication calculations, an individual is capable of directly retrieving answers from pre-existing memory, or alternatively, employing mental calculation strategies based on addition to solve multiplication problems [16]. Logan (1988) also put forward a similar point of view, when a person wants to solve the arithmetic project, the strategy based on calculation and the strategy purely based on memory retrieval will compete with each other, implying that each unique trial is resolved either through retrieval from memory or through an algorithmic operation. Performance is considered automatic when it relies on direct-route retrieval of results from memory, a single-act process. Conversely, performance is considered controlled when it is dependent on algorithmic processing techniques such as counting, adding, memorising, borrowing, or negating a logical term [17, 18].

Some empirical findings in the field of mental arithmetic can also prove the importance of memory retrieval strategies in mental arithmetic. The first one is the problem-size effect, which pertains to the finding that larger arithmetic problems (e.g., 9 + 8) generally take longer time to solve than smaller ones (e.g., 2 + 3) and that the relationship is linear in slope. The assumed reason is that smaller numbers are more frequently used by people. The second one is the tie-effect, which refers to the observation that arithmetic problems involving identical operands (e.g., 4 + 4 or 3 + 3) can be solved more quickly than equivalent non-ties (e.g., 4 + 3 or 3 + 2). Although there is no accepted reason for this, it is true that the sum of two identical numbers is visually more impressive than the sum of two different numbers to remember. The third one is the “carry-over to 10” effect, which relates to the additional cognitive effort required when performing an arithmetic operation that results in a carry-over to the next tens place (e.g., 7 + 6 = 13). The cognitive load increases because of the two-step process: adding the units and then carrying over the extra value to the next tens place [19, 20].

Willis (1992) advocated that mental arithmetic should be the primary form of calculation, with written arithmetic as memory support [21]. However, the facts contradict the idea. Existing research shows that students often neglect learned strategies when solving math problems [22]. For this phenomenon, Blöte et al. (2000) identified the factors affecting their strategy preferences: lack of strategy awareness, weak recognition of strategy value, poor effort management on a strategy, and classroom atmosphere [23]. Considering Hiebert and Wearne’s emphasis on instruction (1996), mathematics instruction may be a key point in children’s choice of numerical strategies [14].

In light of the preceding analysis, this study advocates for an innovative mental arithmetic teaching method. It entails the development of novel instructional aids and a paradigm shift in the teaching method to mathematical mental computation. It also focuses on augmenting the consolidation of mnemonic schemas among primary education students via iterative exercises, and having unadulterated mnemonic retrieval mechanisms take the dominant position during mental calculation tasks, which helps facilitate a progressive shift from algorithmic to mnemonic processing modalities, thereby improving the speed and precision in mental arithmetic practice eventually.

### 2.2. Working memory and schema theory

Cognitive psychology suggests mental arithmetic comprises encoding, arithmetic, and response i.e., encoding information presented externally and then obtaining the answer through internal arithmetic knowledge extraction or arithmetic operations [24]. Hibbert and Lefebvre (1992) explain that primary students’ reluctance to use mental arithmetic is due to difficulty in encoding relationships and inadequate knowledge of placeholder values [25].

Concerning the knowledge base, this paper argues that primary school students lack working memory for some basic arithmetic formulas so that they cannot quickly complete operations that require rounding. And it is suggested that relationships or expressions between numbers be linked to them so that students can quickly extract their working memory when performing arithmetic operations. Difficulty in coding reflects the inability of primary school students to make abstract connections between numbers and understand the relationships and variations between numbers in the arithmetic process. It can be solved through a medium that is easy to comprehend and memorize.

The biggest challenge at this point is to find a numerical training means that can aptly address these two issues. During the encoding stage, information can be input into the information channel in different representations (e.g., visual or auditory forms), and it has been found that differences in the form of input not only have an impact on the encoding stage, but also on the extraction process of mental arithmetic [26]. Different ways of presenting mental arithmetic tasks can trigger different information representations of individuals. And reading arithmetic, compared with the visual arithmetic commonly used in previous teaching, may process the topic information more adequately by inputting it through both visual and auditory representations, which may help children in the lower grades to obtain better performance in simple mental arithmetic [27]. This implies that applying a schema-based approach to teaching can probably build the basis of this desired training means.

Stijn De Rammelaere (1999) pointed out that existing research has revealed two major determinants of mental arithmetic effects, namely, the organization of simple arithmetic facts in long-term memory and the processing of information in working memory. The first one has attracted many researcheres and quite extensive relevant results have been made (e.g., Anderson, 1983). However, the role of working memory in arithmetic has received little attention yet [28, 29].

Working memory is a cognitive system with a limited memory capacity that is used to store information temporarily. But it has important implications for reasoning and guiding decisions and behavior. As part of memory, working memory stores recorded information temporarily so that it can be further included in or compared to long-term memory. It has been shown that working memory is crucial to mental arithmetic as all three components of it—the central executive, the phonological loop, and the visuospatial palette—play a role in mental arithmetic under different conditions. LG Guthrie (2015) explains the relationship more deeply. He put that mental arithmetic tasks often require strategic thinking and thoughtful information processing which needs enough time and effort. Beyond basic, well-rehearsed sums, the calculation is often considered to place a relatively high burden on an individual’s internal resources, such as working memory [30]. Through working memory and relevant executive functions, numbers are deposited, added, and manipulated to solve problems. The load on working memory varies with the complexity of the problem and domain-specific expertise (contribution of long-term memory). To reduce the load on internal resources, cognitive processes migrate to wherever it is easiest to perform “computation” and extend to external resources in dynamically distributed cognitive systems [31]. Individuals’ physical behaviors in their environments are not only integral to distributing the working memory load, but they also provide a scaffolding that allows for the development of new strategies and the expansion of cognitive scope [4, 32,].

Furthermore, it has been pointed out that due to the limited capacity of working memory, people tend to organize recurring or interrelated information into chunks by recoding it in order to expand storage capacity [33]. Research on information chunking first originated in short-term memory, which pointed out that the main function of chunking is to store information [34]. Other research suggests that relative to short-term memory, chunking in working memory has an extra function of temporarily processing of information [35]. Therefore, the study of working memory also necessarily involves the process of chunking information [36]. Schema is exactly a kind of image that chunks and encodes information.

A schema is a cognitive or knowledge structure that exists in memory, abstracted from what the learner has in his or her life [37]. Gilboa (2017) put in his study that schemas are higher-level knowledge structures that organize lower-level representations from long-term memory. As generic reference templates, schemas can be compared with new information, and bind multiple features that appear consistently and simultaneously. Given their non-specific elements, commonalities between experiences can be reflected with considerable overlap and correlation. It should be noted that schemas are dynamic structures that can constantly evolve into new experiences and memories in a process of assimilation and conditioning [38].

The concept of memory as a dynamic, constructive process, with schemas as cognitive organizers, is widely accepted. Schemas are now fundamental in cognitive psychology, used not only in memory research but also for understanding complex cognitive processes.

Concept maps are a type of graph introduced by Novak et al. based on Ausubel’s meaningful learning theory as a mental tool to help learners construct and represent knowledge [39, 40]. They consist of nodes and links representing learners’ understanding of a topic. The key concepts are called nodes. While being connected by different paths and relationships, they form propositions. According to Ausubel’s cognitive assimilation learning theory, meaningful learning occurs when learners organize new knowledge into hierarchies while exploring possible connections between different pieces of knowledge. Elaborative strategies, represented by concept mapping, are dominant in today’s educational psychology community, highly regarded among educators, and considered effective strategies for facilitating meaningful learning [41].

It is clear that schema-based memory efficiently connects elemental representations, aiding in forming working memory and understanding arithmetic relationships in a more visualized and clear way. The paper hypothesizes that a schema-based numerical training method effectively enhances students’ mental arithmetic ability.

### 2.3. Development and application of schema method

In cognitive development theory, Simonton (2022) posits that mental schema plays a vital role in cognitive processes. It is not merely a simple construction of past experiences; rather, it actively filters, screens, and organizes external stimuli in novel contexts, transforming them into a structured modular knowledge framework. By drawing upon existing knowledge and experiences, individuals understand objective objects and obtain the emotional experience of what kind of symbolic significance the objects "should" have and how they "should" be understood [42, 43].

**Fig 1:**
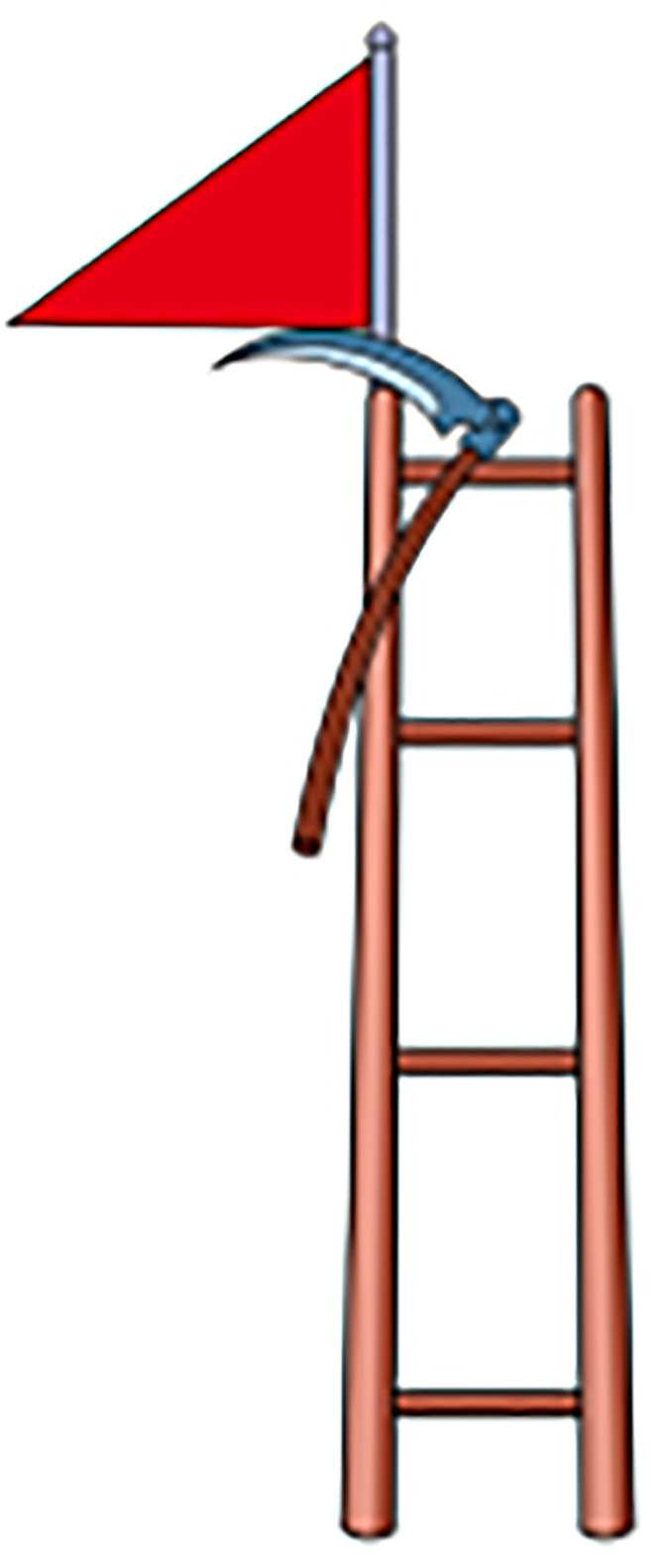
Corresponding schematic teaching AIDS of formula 4+7 = 11.

Based on the above theory, schema-based training aids were devised for subsequent experiments. These aids visually represent the fundamental building blocks of mental arithmetic. The initial step involves encoding numbers into visual symbols or phonetics, like representing "7" as a "sickle," "9" as a "balloon," and "16" as "shí liu" (pomegranate in Chinese). The following step is to merge these coded images into a coherent narrative, focusing on creating a proportional visual representation, where the sum’s image is noticeably larger than the addends’ images., For example, in equation 7+9 = 16, the images for "sickle" and "balloon" are smaller compared to the image for "pomegranate." This step then combines with the story "a sickle from the huge pomegranate dug out a balloon" to interlink these images. The final graphic aids were thus developed. We have developed a total of 45 diagrams (see S1 Appendix) ranging from 1+1 = 2 to 9+9 = 18, covering all single-digit addition equations. In addition, because each graphic aid distinguishes between "addition" and "sum" by the size of the picture, each graphic actually represents four equations, e.g., Fig 1 represents 7+9 = 16, 9+7 = 16, 16–7 = 9, and 16–9 = 7. Compared to traditional methods, schema-based instruction offers lower cognitive strain, increased engagement of students, and a multi-sensory approach integrating kinesthetic sense, five senses, and images characterized by interesting and unique, fostering stronger connections between working memory and long-term memory and transforming working memory to long-term memory, thus improving the retention of memory. Additionally, the approach is more reliable and helpful for addressing persistent errors.

Implementing this form of graphic teaching aids for mental arithmetic is straightforward. First, students familiarize themselves with and memorize the number codes from 1 to 18. Then, the schemas are introduced, encouraging students to create their narratives based on the images to deepen their understanding. Next, relying on the image categories and proportions, the students have to convert the schemas to arithmetic equations. Finally, calculation training was given to enhance students’ proficiency in translating schemas into arithmetic equations and vice versa.

**Fig 2:**
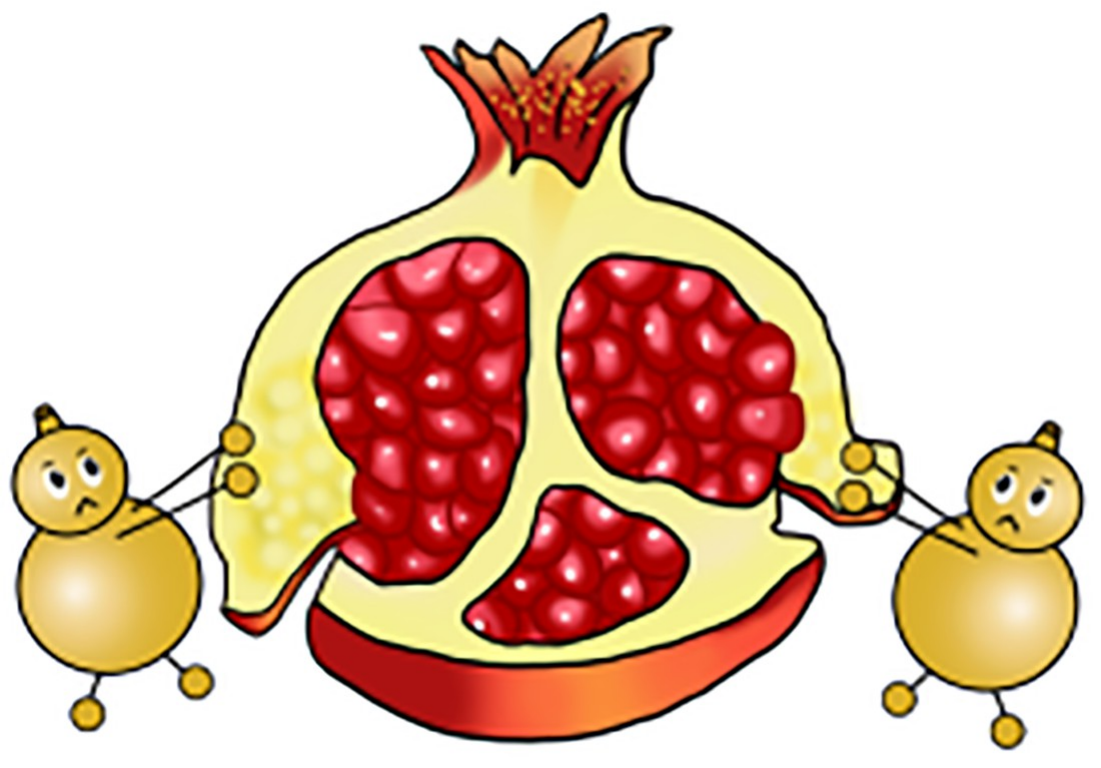
Corresponding schematic teaching AIDS of formula 8+8 = 16.

As shown in Fig 1, the schema uses a red flag, a sickle, and a ladder to represent the numbers "4", "7" and "11", respectively, with the red flag and the sickle geometrically connected to each other acting together on the ladder—representing the equation 4+7 = 11. The schema in Fig 2 has the gourd representing the number "8" and the pomegranate representing the number "16". The two gourds together are connected to the pomegranate—representing the equation 8+8 = 16.

## 3. Research methods

### 3.1. Participants

In this study, the experiment locale was determined by randomly selecting one township from all those within Jingshan County of Jingmen City located in Hubei Province, P.R. China. Subsequently, a single primary school was randomly chosen within this township to serve as the study site, and the third-grade pupils served as the subject source. From this institution, four out of six third-grade classes were randomly selected. Within these selected classes, a cohort of 104 students was randomly invited for participation in the study. These participants were then randomly allocated into either an experimental group or a control group. The control group consisted of 28 male and 24 female students, with a mean age of 9 years. Similarly, the experimental group included 25 male and 27 female students, with the same mean age. The study was conducted in line with the ethical principles of the Declaration of Helsinki and of the American Psychological Association(APA). The study was approved by the ethics committee of the Ethics Professional Committee of Brain Science and Learning Science Committee of Hubei Teachers Education Association(Numbering: S2023-027-2). All procedures were in accordance with the approved guidelines and regulations. Subjects were compensated for their participation.

### 3.2. Procedure

The instruction of mental arithmetic necessitates students to possess not only problem-solving abilities but also proficiency in performing arithmetic calculations swiftly to meet stipulated time standards. In our fieldwork, we observed that a majority of teachers primarily emphasize the accuracy of calculation outcomes during arithmetic instruction, with the requirement for speed being vaguely defined. However, some research demonstrated a significant and positive correlation between mental arithmetic competence and working memory, processing speed, as well as executive function. Overlooking training in processing speed and executive function, as revealed by their study, detrimentally impacts the execution of the mental arithmetic computational process. Insufficient processing speed is manifested in mental arithmetic as delayed response, impaired executive function, and inability to simultaneously memorize and output [44]. Therefore, we adopted both the temporal and accuracy dimensions of mental arithmetic as indicators for gauging mental arithmetic proficiency in our experimentation.

Within this study, mental arithmetic refers to the presentation of questions in a visual form, where the subjects peruse the queries on the test paper and transcribe the answers on the test paper after calculating in their mind without recourse to any penmanship or other computational tools [45]. In order to ensure the accurate reflection of students’ mental arithmetic ability, the students are required to write down the answers as promptly as possible, thereby confirming their utilization of working memory within their long-term memory framework.

The experiment design followed a pretest and post-test structure employing the pre-addition and pre-subtraction scales, drawn from the Center for Brain Science and Learning Power Research of Hubei Second Teachers College. Each of these scales encompassed 44 addition test questions and 44 subtraction test questions, respectively, each scales incorporated an exhaustive set of single-digit addition or subtraction questions. The sequence of these questions was systematically randomized to ensure a non-repetitive and scrambled arrangement, precluding any duplication of mathematical expressions. detailed in the S2 Appendix.

Prior to the commencement of the experiment, a pretest was administered to both the experimental and control groups, with the time taken and error rates in addition and subtraction tests recorded as baseline data. Research suggests that under instructions emphasizing speed, students are more likely to employ memory retrieval strategies for arithmetic tasks [46]. Therefore, during testing, participants were also instructed to “calculate at their fastest speed”, thereby ensuring the predominant use of such strategies. In post instruction, a controlled experimental approach was adopted. Over a continuous period of 14 days, within the control group, assistant teachers organized supervised traditional calculation drills, encompassing 20-minute sessions of one- and two-digit addition and subtraction practice. Next, an additional 10-minute period was allocated for the explanation and analysis of common error-prone problems with pre-recorded online lessons. Conversely, students in the experimental group watched a 20-minute instructional video on the schema-based mental arithmetic method uniformly every day for the first 7 days. The video provides a vivid analysis of each set of computational schema composition, followed by a 10-minute review of previously covered schemas each time, with the help of the pre-recorded instructional video. The videos were consistently presented by the same volunteer teacher to avoid variances out of teacher in teaching. The last 7 days consisted of 20-minute sessions of one- and two-digit addition and subtraction arithmetic exercises, which required students to associate the corresponding schemas when solving the problems. Following 14 days of training, the mental arithmetic skills of both control and experimental students were tested on the 15th day, by means of uniform addition and subtraction post-test scales. Similarly, students were instructed to work out the results as quickly as possible, with their single test duration and scores being recorded as post-test data. To maintain experimental integrity, the experimental and control groups were segregated into different classrooms for the post-tests, each with teacher supervision to prevent inter-group communication. This segregation effectively concealed perception of group differences among the participants, ensuring a placebo effect in the experimental design.

### 3.3. Data collection and analysis

Each experiment requires the integrated coordination of elemental acts. The rationale proposed by the Individual Differences in Response Time (IDRT) model [47] and the multitrait—multimethod (MTMM) approach [48, 49] was applied as a heuristic method to assess the convergent and discriminant validity of competing performance measures. Subsequently, following the completion of each test, a descriptive statistical analysis was initially conducted with Excel tool for response times (RT) and error percentages (ER) of each participant in both the experimental and control groups. Then, with the help of SPSS software, the descriptive statistics for pretest and post-test data of the two groups were computed, encompassing measures such as the average response time (RTM), average error rate (ERM), variance (SD), skewness (Skew), and the Mann-Whitney U test was conducted to derive the corresponding p-values. Following this, leveraging the descriptive statistical data, we utilized Python to compute the correlation between pretest and post-test RT and ER, thereby establishing a Multitrait-multimethod-matrix to evaluate the reliability, as well as convergent and discriminant correlations, of the alternative performance indices in the self-paced speed tests.

## 4. Results

### 4.1 Descriptive statistical analysis of the experimental and control groups

As depicted in Table 1, the addition test duration was 103.8 seconds for the control group and 103.1 seconds for the experimental group. These two means demonstrate a high degree of proximity, and the Mann-Whitney U test indicated that there was no significant difference between the results of the two groups (p = 0.899>0.05). Likewise, the subtraction test time was 104.2 seconds for the control group and 104.9 seconds for the experimental group. Once again, no significant difference was evident (p = 0.534>0.05). Therefore, there exists no significant difference between the control and the experimental groups in terms of the time spent on addition and subtraction tests.

**Table 1:** Descriptive statistical analysis of pretest and post-test durations.

RT		Session 1					Session 2				
		M	SD	Skew	Range	p	M	SD	Skew	Range	p
Addition	C	103.8	11.9	-0.3	78~123	0.899	94.7	8.2	0.2	71~118	0.003
	E	103.1	12.9	-0.2	78~122		87.7	15.6	0.1	64~114	
Subtraction	C	104.2	12.6	0.5	85~128	0.537	98.6	14.2	0.2	71~130	0.044
	E	104.9	12.3	-0.3	78~126		91.8	15.7	0.0	62~126	

Population parameters for all performance measures. N = 52 (experimental group); N = 52 (control group); Session 1, pretest; Session 2, post-test; RT, response time; C: control group; E: experimental group.

In contrast to the pretest findings, a significant difference emerged in the post-test durations between the experimental and control groups. Specifically, the mean addition post-test duration was 94.7 seconds for the control group and 87.7 seconds for the experimental group, resulting in a significant difference of 7.02 seconds (p = 0.003<0.05). Similarly, the mean subtraction duration was 98.6 seconds for the control group and 91.8 seconds for the experimental group, showing a significant gap of 6.8 seconds (p = 0.044<0.05). For the same set of problems, a shorter time taken indicates a faster mental calculation speed. Participants who underwent schema training exhibited superior speed in both addition and subtraction compared to the control group.

The data were analyzed from the perspective of standard deviation. The standard deviation of the addition and subtraction pretesting time of the control group was 11.86 and 12.59 respectively, and that of the experimental group was 12.86 and 12.31 respectively. This indicates a comparable level of dispersion between the two groups. On the other hand, the standard deviation of addition and subtraction post-testing time of the control group was 8.16 and 14.22 respectively, and that of the experimental group was 15.59 and 15.73 respectively. It can be concluded that there is a greater difference in the testing durations of students trained with the schema teaching method compared to the traditionally trained students, which might be closely related to learning efficiency, attitudes during the learning process, and adaptation to the novel instructional approach.

Table 2 presents the differences in error rates between pretest and post-test assessments for the control and experimental groups. As indicated in the tables, there were no significant differences in pretest error rates for addition between the two groups (p = 0.073>0.05). Similarly, no significant differences were observed in pretest error rates for subtraction (p = 0.243>0.05). Thus, there were no significant differences in error rates for addition and subtraction pretests between them. In contrast, the mean error rate for addition post-tests in the control group was 0.08, while the experimental group exhibited a mean error rate of 0.01, indicating a significant difference (p = 0.00*<0.05). For subtraction post-tests, the control group had a mean error rate of 0.05, whereas the experimental group had that of 0.03, also showing a significant difference (p = 0.00*<0.05). This suggests that students in the experimental group, who underwent schema training, demonstrated higher accuracy in both addition and subtraction compared to the control group.

**Table 2:** Descriptive statistical analysis of the pretests and post-test scores.

ER		Session 1					Session 2				
		M	SD	Skew	Range	p	M	SD	Skew	Range	p
addition	C	0.13	0.08	-0.37	0.0~0.24	0.073	0.08	0.04	-0.48	0.0~0.14	0.000
	E	0.11	0.06	-0.32	0.0~0.2		0.01	0.01	0.39	0.0~0.04	
subtraction	C	0.12	0.04	-0.88	0.0~0.18	0.243	0.05	0.04	0.19	0.0~0.14	0.000
	E	0.13	0.05	-1.09	0.0~0.18		0.03	0.03	0.35	0.0~0.1	

Population parameters for all performance measures. N = 52 (experimental group); N = 52 (control group); Session 1: pretest; Session 2: post-test; ER: error percentage; C: control group; E: experimental group.

From the perspective of standard deviation, statistical analysis of the standard deviations of error rates in addition and subtraction tests for the control and experimental groups revealed noteworthy patterns. On one hand, the standard deviations for all post-tests were smaller than those for pretests, indicating a reduction in the variability of error rates among students after the assessments. On the other hand, the standard deviation for addition post-tests in the experimental group (0.01) was significantly smaller than that in the control group (0.06). Similarly, the standard deviation for subtraction post-tests in the experimental group (0.03) was smaller than that in the control group (0.05). These results suggest that experimental group exhibited lower variability in error rates and its students showed greater stability in mental calculation accuracy compared to their counterparts in the control group.

The aforementioned findings underscore a significant increase in students’ calculation speed and accuracy after training of the schema teaching method. This improvement can be attributed to the achievement of automatization in arithmetic skills. An individual can directly extract the answers to arithmetic problems from long-term memory without conscious cognitive manipulation, a process known as automated extraction [50, 51]. Arithmetic units composed of basic arithmetic knowledge that has been repeatedly practiced and experienced to the extent of automated extraction [18, 52] transform into arithmetic automata [53].

In summary, comparing the results of test duration and test error rates, it is evident that the schema teaching method is more conducive to enhancing students’ mental calculation abilities. Regarding test duration, the effectiveness of the method is pronounced, as the experimental group took significantly shorter testing times compared to the control group after training. In terms of test error rates, there were significant differences between the post-test error rates of the experimental group and the control group, with the former performing better than the latter. Therefore, the schema-based calculation training method is of the potential to accelerate, improve, and stabilize learners’ mental arithmetic abilities.

### 4.2 Correlation analysis of the experimental and control groups

#### Retest reliability

Reliability coefficients are presented on the main diagonal of the correlation matrix (Table 3), illustrating the correlations between pretest and post-test administrations. The reaction time (RT) for addition in the control group (r = 0.886), subtraction in the control group (r = 0.983), addition in the experimental group (r = 0.980), and subtraction in the experimental group (r = 0.991) demonstrate high reliability. Additionally, the error rates (ER) for addition in the control group (r = 0.967), subtraction in the control group (r = 0.907), addition in the experimental group (r = 0.854), and subtraction in the experimental group (r = 0.860) exhibit good reliability. It is noteworthy that considering the generally low or insufficient reliability of error scores in most mental calculation tasks (Maloney et al., 2010; Steinborn et al., 2016, 2018; Hedge et al., 2018, for an overview), the obtained reliabilities for error rates are substantial [54–56].

**Table 3:** Multitrait-multimethod-matrix for control group and experimental group.

		Session 2	Session 1	
			RT	ER
**C**	**addition**	RT	0.886	-0.054
		ER	0.085	0.967
	**subtraction**	RT	0.983	-0.006
		ER	-0.060	0.907
**E**	**addition**	RT	0.980	0.191
		ER	-0.009	0.854
	**subtraction**	RT	0.991	0.056
		ER	-0.024	0.860

Test-retest reliability and inter-correlation structure (convergent vs. divergent) of all perfor-mance measures, separately for session 1 (pretest) and session 2 (post-test). E: experimental group; C: control group; N = 52 (experimental group); N = 52 (control group); RT: response time; ER: error percentage. Test-retest reliability is shown in the main diagonal (denoted with gray); correlations for the first session are shown above, for the second session below the main diagonal.

#### Correlational structure

In the tests for addition and subtraction in both the control and experimental groups, the correlations between average response time (RTM) and error rate (ER) are consistently low, with correlation coefficients (r values) generally below 0.1 (Table 3). This suggests a lack of association between RTM and ER during mental calculation processes. This conclusion further supports the notion that the schema-based mental arithmetic training method enables the learners to enhance both their calculation speed and accuracy.

Obviously, the schema teaching method effectively solves the problems faced by students during mental arithmetic exercises. Additionally, it allows students to transcend reliance on pen-and-paper mental image calculation. The advantage of graphical teaching lies in the seamless integration of multiple encoded graphs, forming a coherent memory structure, which is exactly the right match for the two-digit addition and subtraction involving three elements, consequently leading to notable enhancements in calculation speed and accuracy, thus achieving instructional objectives. The outcomes of this test confirm the conjecture presented in this paper that the schema training method is an effective training method for augmenting students’ mental arithmetic ability.

At the end of the post-test, a survey was conducted to ascertain the calculation strategies adopted by students in the experimental group during the test. An overwhelming 83% of the students responded that they employed the “schematic coding" approach independently, while only 17% continued to use the method of "working with numbers in their head". This finding implies that this study’s teaching method, rooted in schemas, effectively addresses students’ inclination to circumvent the utilization of mental arithmetic strategies.

## 5. Conclusion and prospect

### 5.1 Conclusion

In response to the current state of mental arithmetic practice of elementary school students, numerous scholars have suggested that children should be encouraged to develop their own mental arithmetic strategies [57]. However, in practice, teachers and students often equate "computational ability" with "computational skills," failing to create effective and generally accessible mental arithmetic strategies [14]. This study conducts experimental research with schema encoding viewed as a memory support for mental arithmetic expressions.

The results of the study robustly demonstrate a significant improvement in students’ mental arithmetic abilities through schema-based mental arithmetic training. In both addition and subtraction tests for the control and experimental groups, schema training exhibited out-standing effectiveness in terms of Response Time (RTM) and Error Rate (ER) (in the addition and subtraction tests, with the pretest RTM almost being equal between the control and experimental groups, reduction of the post-test duration time in the experimental group was 1.69 times that of the control group for addition and 2.34 times for subtraction. In terms of ER, with the pretest ER almost being equal between the two groups, the post-test ER reduction in the experimental group was 2 times for addition and 1.43 times for subtraction compared to the control group). Additionally, the stability and reliability of the results was confirmed. These findings indicate together that through schema-based teaching, students can effectively enhance mental arithmetic speed and accuracy, overcoming the issues of speed and accuracy faced in traditional mental arithmetic processes.

The low correlation between RTM and ER in both the control and experimental groups further supports the effectiveness of schema training. This suggests that students under schema training can independently use schema encoding to obtain answers, other than overly relying on traditional pen-and-paper mental imagery algorithms. This not only enhances the efficiency of mental arithmetic but also indicates that schema training enables students to confidently tackle mental arithmetic tasks, lifting them out of dependence on external tools.

### 5.2 Contributions and significance of the study

The major contribution of this research lies in revealing the outstanding effects of schema-based mental arithmetic training in improving students’ mental arithmetic speed and accuracy. Systematic experimentation demonstrates that schema training is not only theoretically feasible but also yields significant educational outcomes in practical applications. This discovery provides an innovative and viable teaching method for the field of mental arithmetic education, simultaneously offering students a more effective learning path.

The significance of the study extends beyond uncovering the positive impact of schema training on mental arithmetic abilities; it also provides an innovative teaching method for mental arithmetic education. Traditional mental arithmetic teaching often neglects students’ avoidance of mental arithmetic strategies, but this study effectively addresses this issue through schema-based teaching, offering new perspectives for the future development of mental arithmetic education. Future research and practice can draw upon the teaching method employed in this study to further explore the applicability of schema training in different backgrounds and conditions, better serving the learning needs of diverse students, eventually to make a positive contribution to driving innovative development in the education sector.

### 5.3 Recommendations

Based on the outcomes of this research, it is recommended to actively promote schema training as an effective means of mental arithmetic education in schools and educational institutions. By leveraging modern technological tools, such as smartphone applications and online learning platforms, to integrate schema training into digital education, teaching flexibility and accessibility can be enhanced, allowing more students to benefit from schema training. Educators can expand the adoption of this innovative teaching method by providing relevant training and teaching resources.

It has to be admitted, this experiment has its limitations. For instance, the sample in the experiment is relatively small, with the participants concentrated in rural schools in a specific region. Subsequent research can expand the source and increase the quantity of participants, and prolong the testing period for further validation. Further investigation into the applicability of schema training across different age groups and academic disciplines is warranted, which would contribute to a more comprehensive understanding of the educational potential of schema training and provide guidance for tailoring more specific teaching strategies. Additionally, exploring whether students trained with schema can exhibit outstanding performance in mathematical application abilities, innovation, and metacognition should also be a subject for further exploration.

## Supporting information

S1 Data (XLSX)

S2 Data (XLSX)

S1 Appendix Appendix A. (DOC)

S2 Appendix Appendix B. (DOC)
