# Supplementary figures and images for: Improving mental arithmetic ability of primary school students with schema teaching method: An experimental study
Source: PLoS One. 2024 Apr 16;19(4):e0297013. doi: 10.1371/journal.pone.0297013 (PMC11020525; doi:10.1371/journal.pone.0297013)

**Appendix A**

| 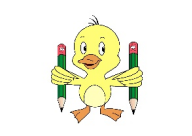 | 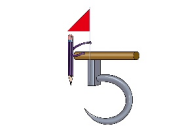 | 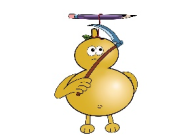 | 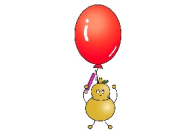 |
| --- | --- | --- | --- |
| 1+1=2 | 1+4=5 | 1+7=8 | 8+1=9 |
| 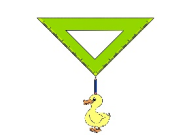 | 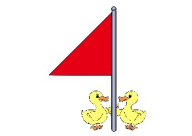 | 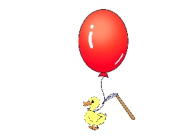 | 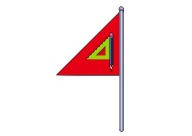 |
| 1+2=3 | 2+2=4 | 2+7=9 | 1+3=4 |
| 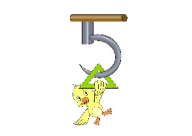 | 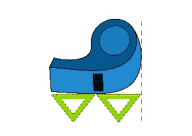 | 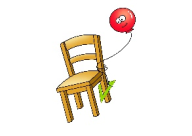 | 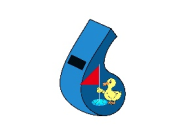 |
| 3+2=5 | 3+3=6 | 3+9=12 | 4+2=6 |
| 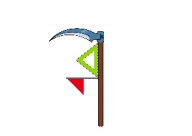 | 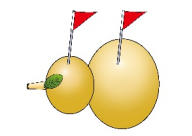 | 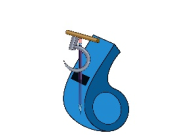 | 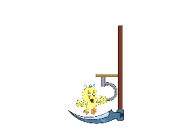 |
| 4+3=7 | 4+4=8 | 5+1=6 | 5+2=7 |
| 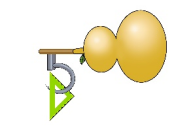 | 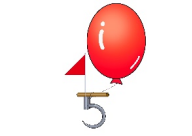 | 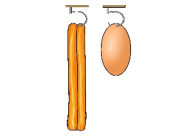 | 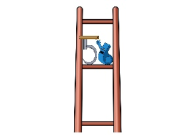 |
| 5+3=8 | 5+4=9 | 5+5=10 | 5+6=11 |
| 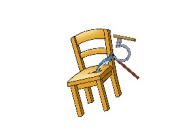 | 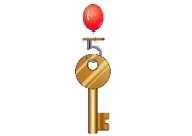 | 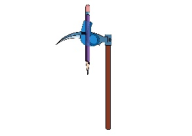 | 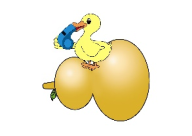 |
| 5+7=12 | 5+9=14 | 6+1=7 | 6+2=8 |
| 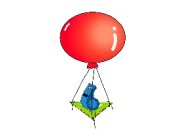 | 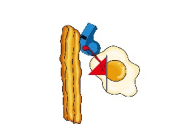 | 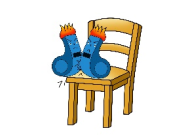 | 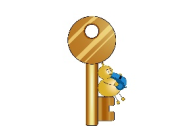 |
| 6+3=9 | 6+4=10 | 6+6=12 | 6+8=14 |
| 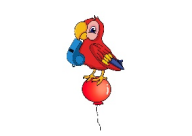 | 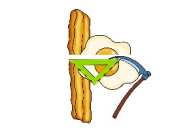 | 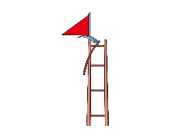 | 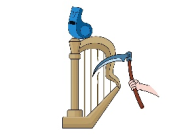 |
| 6+9=15 | 7+3=10 | 7+4=11 | 7+6=13 |
| 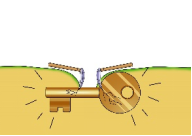 | 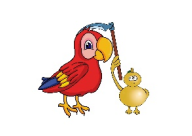 | 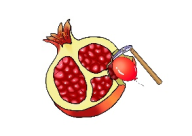 | 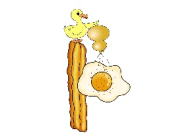 |
| 7+7=14 | 7+8=15 | 7+9=16 | 8+2=10 |
| 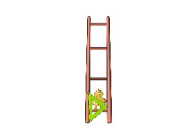 | 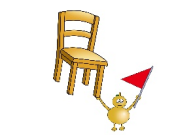 | 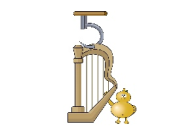 | 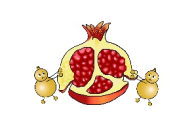 |
| 8+3=11 | 8+4=12 | 8+5=13 | 8+8=16 |
| 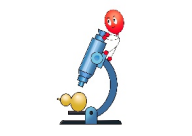 | 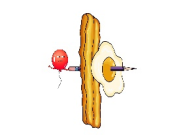 | 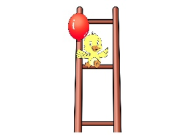 | 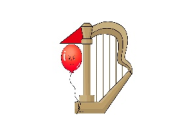 |
| 8+9=17 | 9+1=10 | 9+2=11 | 9+4=13 |
| 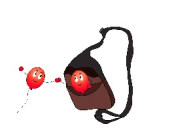 |  |  |  |
| 9+9=18 |  |  |  |

Supplement: S1 Appendix — (DOC) [file pone.0297013.s003.doc]

**Appendix B**


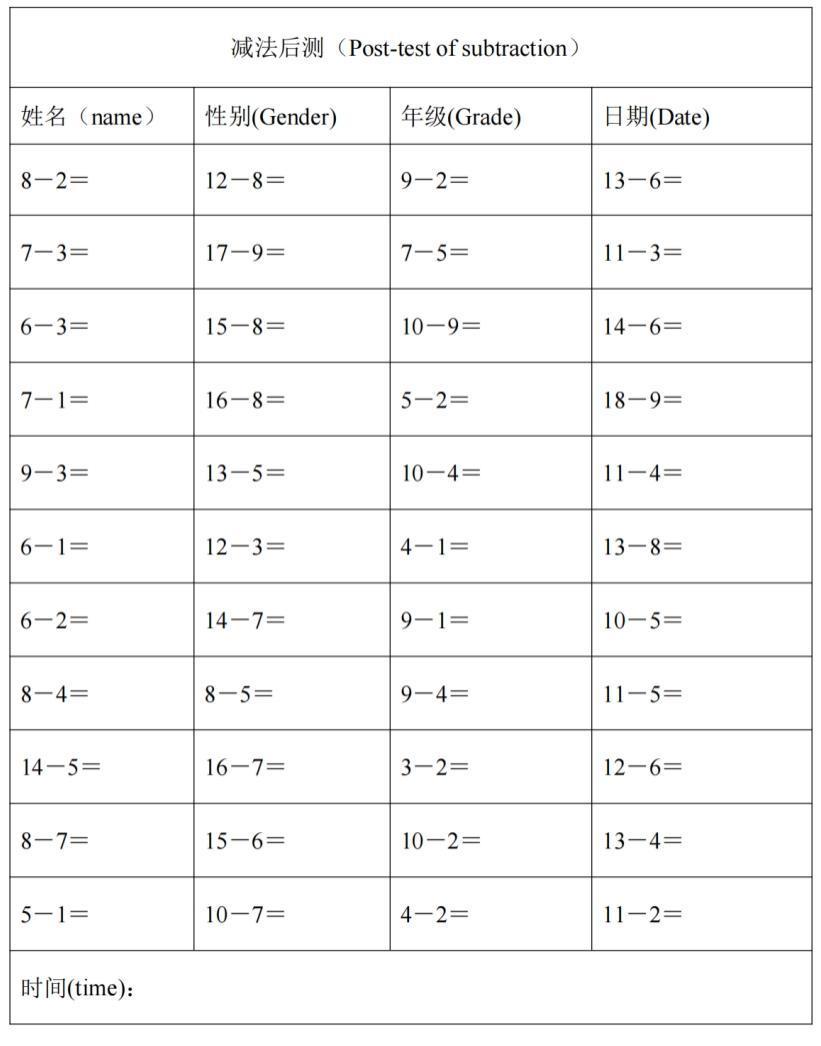

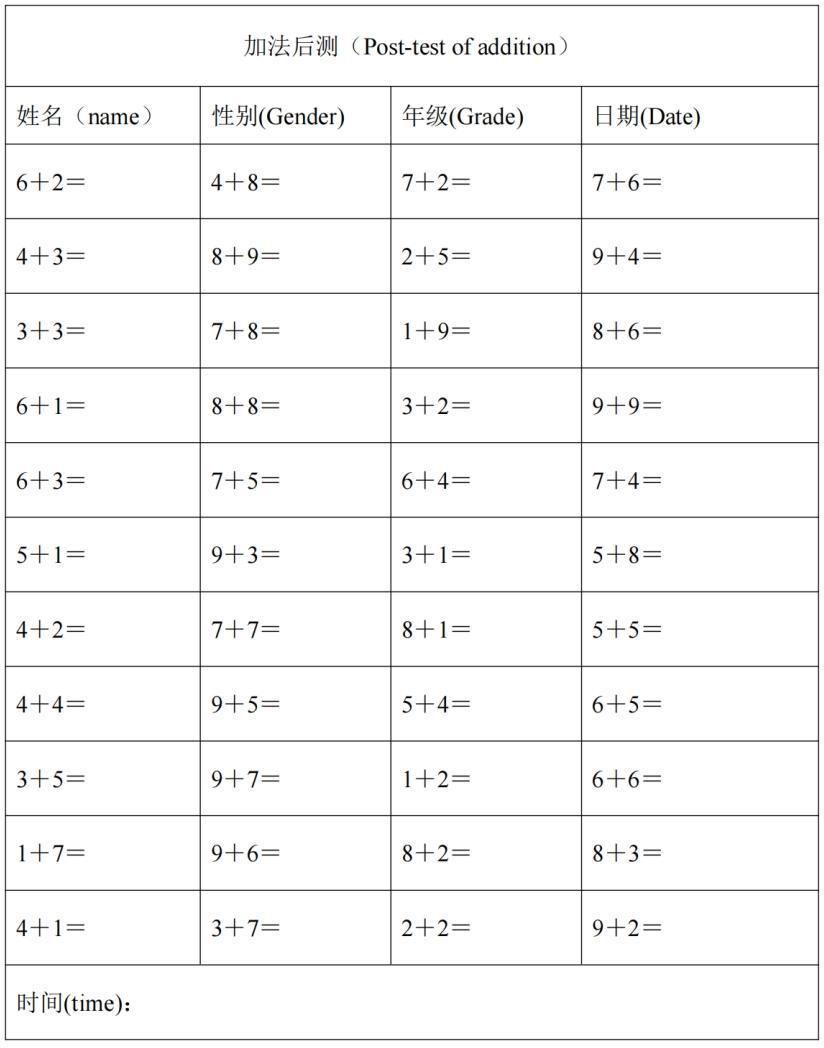


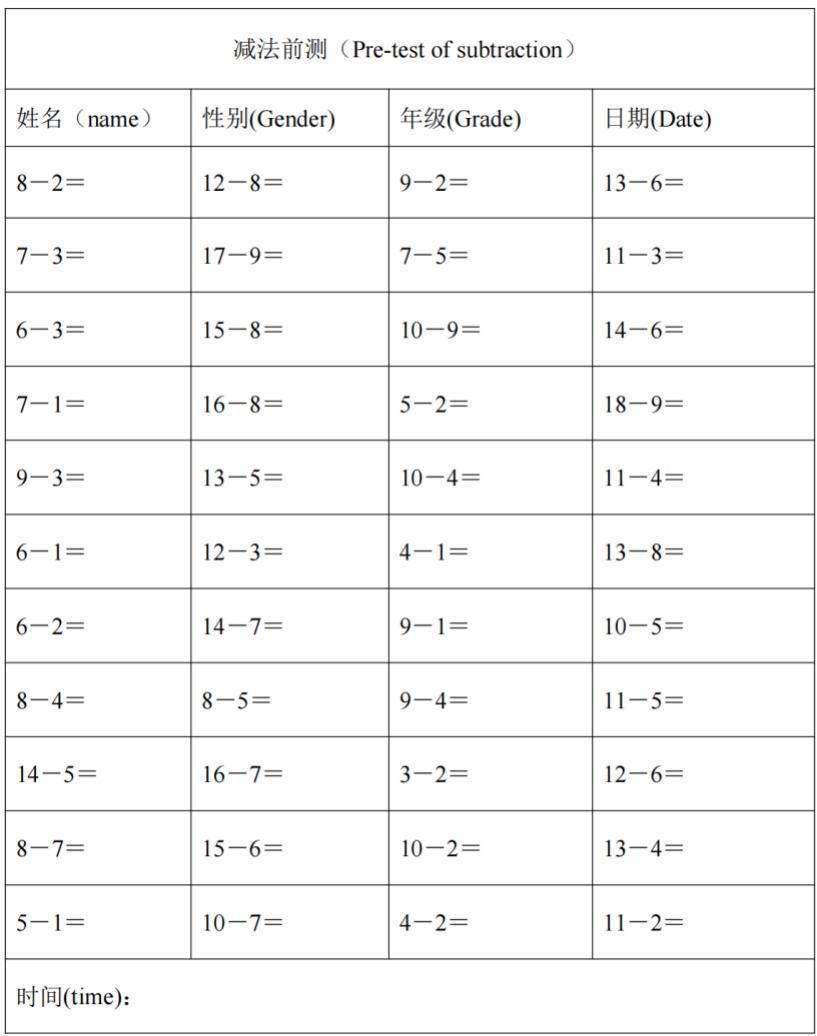

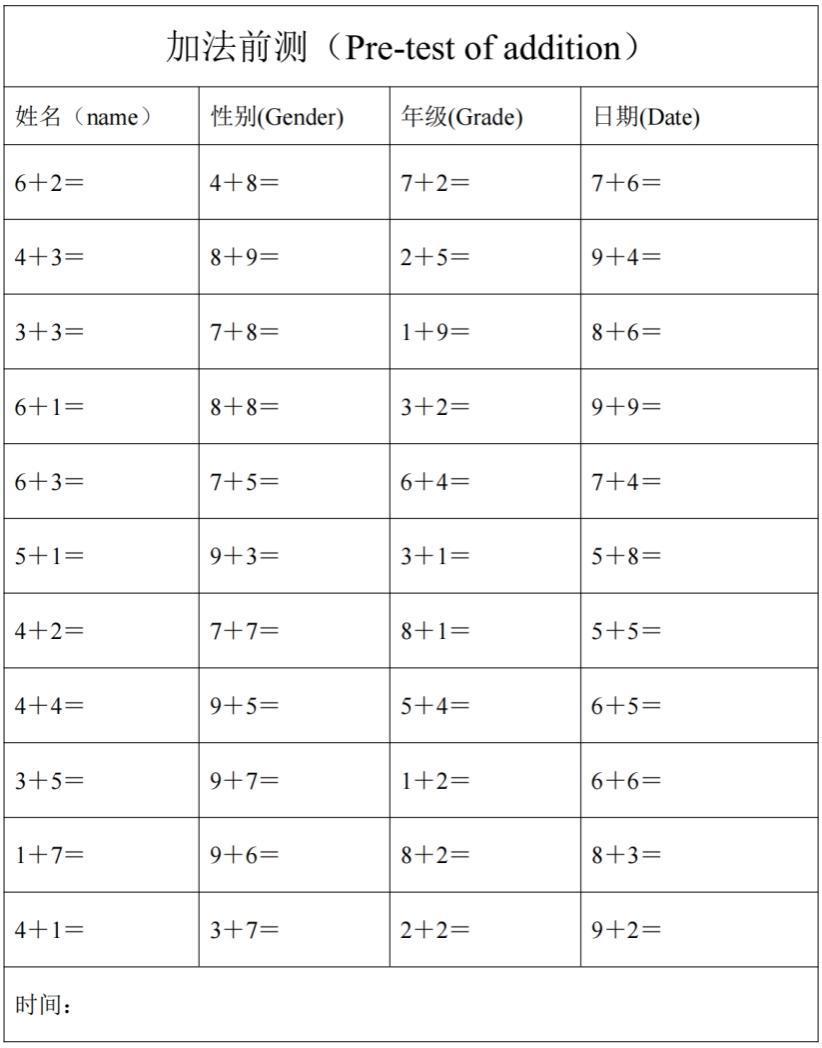

Supplement: S2 Appendix — (DOC) [file pone.0297013.s004.doc]
